# Nocturnal at Noon? Addressing False Positives in Activity Pattern Data

**DOI:** 10.1002/ece3.73388

**Published:** 2026-03-30

**Authors:** Mohamed Khalil Meliane, Nicole Rita, Zachery B. Holmes, Joseph M. Guthrie, E. Hance Ellington

**Affiliations:** ^1^ Range Cattle Research and Education Center University of Florida Ona Florida USA; ^2^ Department of Wildlife Ecology and Conservation University of Florida Gainesville Florida USA; ^3^ Archbold Biological Station Venus Florida USA

**Keywords:** automated wildlife detections, Bayesian model, bioacoustic monitoring, circadian rhythm, computer vision, misclassification

## Abstract

False positives in wildlife detection datasets can substantially bias ecological inference, including diel activity pattern estimation from time‐stamped detections. This problem is intensifying with widespread use of automated classifiers for camera‐trap images and bioacoustic recordings, where full human verification is rarely feasible. Here, we present a Bayesian framework that provides a false‐positive‐aware alternative to commonly used pooled kernel density estimation workflows for diel activity. The model uses a subset of human‐verified records to estimate (i) θ, the dataset‐average true‐positive rate among apparent detections, and (ii) time‐of‐day distributions describing the relative frequency of true detections and false positives across the 24‐h cycle. We implement two error structures: a parsimonious uniform false‐positive model and a flexible “skewed” model that learns a false‐positive diel distribution to accommodate temporally clustered misclassifications. Simulations across multiple activity shapes, false‐positive rates, and verification intensities showed that the approach can recover true diel patterns with limited verification effort and reduces bias relative to uncorrected estimates. We illustrate the method on an empirical dataset, where accounting for false positives yielded meaningfully different activity inferences than standard pooled approaches. Finally, we discuss extensions that allow θ to vary with covariates or random effects (e.g., site/night) and highlight opportunities to integrate diel activity correction with modern false‐positive occupancy and abundance models.

## Graphical abstract

We present a hierarchical Bayesian framework that corrects activity pattern estimates from datasets containing false‐positive detections. We show that our method recovers true activity patterns with high fidelity across multiple activity shapes, false‐positive rates and verification efforts.

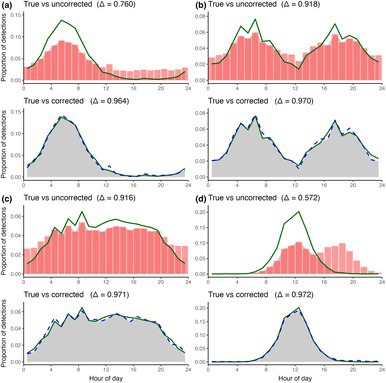

## 1 Introduction

Artificial intelligence (AI) is accelerating productivity across numerous sectors. The ability of ecologists to benefit from the tool is, however, still hampered by methodological gaps (Wright et al. 2020; Gewin 2025). Primarily, the increasing availability and adoption of AI computer vision tools to label ecological data ranging from camera images to bioacoustic records of birds, bats, and other vocal taxa has heightened concerns about false positives. While historically considered rare due to expert‐led data collection, methodological advances, particularly in spatial analyses, have revealed that false positives can substantially distort ecological inference (Miller et al. 2011). Coupled with automated data collection tools, models such as BirdNET (Kahl et al. 2021), which identifies species from audio recordings, MegaDetector (Beery et al. 2019), and SpeciesNet (Gadot et al. 2024), which classify animals in camera trap images, now process data at unprecedented scales. However, the sheer volume of their output is rapidly exceeding the capacity of human verification, positioning false positives as a major challenge for the future of ecological data analysis and underscoring the need for adequate methods.

False positives are relatively well studied in spatial distribution analyses where an observer can mistake a species' call or appearance for another's, leading to biased occupancy (Miller et al. 2011; Chambert et al. 2015) or abundance estimates (Chambert et al. 2016). Early solutions explicitly modeled misidentification to reduce these biases and improve inference (e.g., Royle and Link 2006; Miller et al. 2011; Chambert et al. 2016), and subsequent work has emphasized how accounting for misclassification can substantially enhance spatial ecological estimates (Rojas et al. 2019; Manica et al. 2019). More recently, a growing class of models has extended this false‐positive framework to automated acoustic and other sensor data, integrating detection outputs with auxiliary information (e.g., manual validation subsets and/or complementary surveys) to estimate occupancy, abundance, and trends while accounting for misclassification and detection heterogeneity (Doser et al. 2021; Clement et al. 2022; Udell et al. 2024).

Despite its importance, there is currently no widely adopted framework for correcting false positives in diel activity pattern estimation. For instance, shifts in species' activity patterns are used to measure responses to predators, environmental stress or seasonal patterns (e.g., forest mammal activity varies with thermoregulation and predator avoidance; Vallejo‐Vargas et al. 2022). In other cases, changes in activity patterns following human presence can indicate anthropic disturbance (e.g., mammals become more nocturnal near humans; Gaynor et al. 2018). AI models use common patterns to identify species, making them susceptible to errors when species with some similarities co‐occur. For example, BirdNET is known to frequently confuse Boreal Owl (*Aegolius funereus*) calls with those of Wilson's Snipe (*Gallinago delicata*) generating false nocturnal detections, such misclassifications could result in misleading estimates of activity pattern (Ware et al. 2023).

In this paper, we present a hierarchical Bayesian modeling framework that uses a subset of human‐verified detections within a larger AI‐labeled dataset to enhance diel activity pattern estimates in the presence of false positives. The verified subset informs the model about the distribution of true detections and enables the differentiation of genuine activity signals from misclassifications. Using simulations and an empirical dataset, we show that explicitly accounting for false positives reduces bias and can substantially improve inferred diel activity patterns relative to standard uncorrected estimates.

## 2 Methods

### 2.1 Model Descriptions

We formulated both methods as non‐parametric, histogram‐based Bayesian mixture models, implemented in RStan (Stan Development Team 2025). In each case, the day is divided into *J* equally spaced time‐of‐day bins (*J* = 24 for the 1‐h bins used in the following examples), and each detection is allocated to a bin *b*. A latent indicator *y*_i_ denotes whether detection *i* is a true positive (species present) or a false positive (misclassification). A subset of observations is verified by an expert observer, providing ground truth labels that inform the true positive rate and the weights of the time bins.

Let *f* denote a time‐of‐day distribution: it is a set of 𝐽 non‐negative values that sum to 1, where *fj* is the proportion of detections expected to occur in time bin 𝑗. Thus, *f* describes the shape of the activity pattern across the day as the relative frequency across time. We use θ to denote the true‐positive rate among AI‐labeled records. In practice, θ is informed by the verified subset and can be interpreted as the species‐specific‐average fraction of detections that are truly the target species. For example, if 200 AI‐labeled records are checked by an expert and 150 are confirmed as the target species, then θ = 0.75 for that dataset. In both models, θ governs how much of the observed data are assigned to true vs. false detections, while *f* governs when those detections occur across the day. Under a pooled analysis, θ is assumed constant across records; if users suspect that θ meaningfully varies within the dataset, θ can be extended to include covariates and/or random effects (see Section 4).

#### 2.1.1 When False Positives Are Uniformly Distributed

Under this scenario and to model the posterior mean that is, true activity pattern *f*, we assume that false positives occur uniformly across the 24‐h cycle, each of the time bins is assigned an error probability of 1/*J*. To minimize the model's assumptions, we use a flat Dirichlet prior to indicate that the model initially assumes that activity is equally likely in each time bin. Similarly, the true positive rate θ is assumed to follow a Beta (1,1) distribution indicating that all values are initially equally likely. We then use verified and unverified records as follows:

1. For verified records: If *y*_*i*_ = 1 (correct ID), the detection increases the weight of its respective time‐bin in the activity histogram *f*. When *y*_*i*_ = 0 (verified false positive), we treat the detection as a misclassification.
2. For unverified records we use a two‐component mixture (Equation 1) to assign a likelihood depending on their respective timestamp:

(1) $$p\left(\mathrm{bi}|\theta ,f\right)=\theta f_{\mathrm{bi}}+\left(1-\theta \right)\frac{1}{J}$$

where *f*_bi_ is the current estimate of the species' probability of being active in that bin. 1/*J* describes the uniform distribution of false positives. For example, 1/*J* = 0.04 if 24 hourly bins are modeled. *θ* true positive rate learned from the verified data.

#### 2.1.2 When False Positives Are Skewed

To capture scenarios where misclassifications are temporally clustered (e.g., AI errors concentrated at night), we extend the mixture to include a second error histogram *f*_false positive_. Similar to the uniform error model, both *f*_true_ and *f*_false positive_ receive Dirichlet (1*J*) priors and θ retains a Beta (1,1) prior. Here, verified records of true detections update *f*_true_ while verified false positives inform *f*_false positive_.

For unverified records the mixture likelihood (Equation 2) for each record is calculated as follows:

(2) $$p\left(\mathrm{bi}|\theta ,f_{\text{true}},f_{\text{false positive}}\right)=\theta f_{\text{true},\mathrm{bi}}+\left(1-\theta \right)f_{\text{false positive},\mathrm{bi}}$$

where *f*_true_ is the current estimate of the species' probability of being active in that bin. *f*_false positive_ replaces the uniform 1/*J* probability from the previous model and is the probability of the false positive occurring in that time bin. θ true positive rate learned from the verified data.

This two‐histogram mixture allows both the shape and the timing of erroneous detections to be learned from the data, rather than assuming a uniform background.

#### 2.1.3 Links to Spatial False‐Positive Informed Models and Kernel Density Estimation of Diel Activity

Our diel activity model is focused on estimating a species' time‐of‐day activity pattern in the presence of misclassification. Nevertheless, the likelihood structure is set within a framework that is comparable to observation and misclassification components of false‐positive models developed for occupancy and abundance estimation. In false‐positive occupancy models, observations are commonly represented as a mixture that allows detections to arise either from true presence or from false positives due to misidentification (Royle and Link 2006). Similarly, false‐positive N‐mixture and integrated acoustic‐abundance models extend this idea to abundance by combining a latent abundance/detection process with a misclassification process and using a subset of manually validated detections to learn error rates (Doser et al. 2021; Udell et al. 2024).

Our formulation can be viewed as a record‐level mixture analogue of these models, conditioned on the set of AI‐labeled records used to construct the diel curve. Each record is assumed to be either a true detection or a false positive. The parameter θ is informed by the verified subset in the same spirit as “auxiliary validation” data in false‐positive occupancy/N‐mixture models (Doser et al. 2021). The distributions *f*_true_ and *f*_false_ then describe when true detections and false positives occur across the 24‐h cycle (relative frequency across time bins).

The main goal of our approach is to provide a false‐positive–aware alternative to the kernel density estimation workflows widely used to estimate diel activity. We therefore estimate a population‐averaged activity pattern by pooling detections across sampling units and occasions, mirroring common kernel applications in which a species' activity curve is derived from a vector of detection times (Ridout and Linkie 2009; Rowcliffe et al. 2014). Under this pooled estimate, θ is treated as a constant parameter representing the dataset‐average (record‐weighted) prevalence of true detections among all detections used to construct the activity curve. Complementarily, *f*_false_ captures when false positives occur across the 24‐h cycle, which is critical for correcting spurious peaks arising from temporally structured misclassifications. When site, night, or other covariates are retained and heterogeneity in prevalence is a concern, θ can be extended using a logit link with covariates and/or random effects; we describe practical use cases for this extension in the discussion.

### 2.2 Simulation Study

We evaluated both models on simulated datasets designed to mimic common activity patterns and error processes. For comparability, we fixed the total number of detections and set θ to known values in simulations. In ecological applications, both the total number of detections and θ vary as functions of abundance, detection conditions, and misclassification with non‐target species (see Section 2.1.3 for more information).

#### 2.2.1 Uniform‐Error Scenario

For this scenario, we proposed that true patterns follow three shapes that could be encountered in nature: unimodal, bimodal, and trimodal. We drew values from von Mises distributions to simulate peaks at (𝜋/2), (𝜋/2, 3𝜋/2), and (π/2, π, 3π/2 rad), respectively. We injected a 0.30 false positive rate uniformly across the day (0, 24) to simulate a uniform error pattern. We defined the total number of detections *N* = 4000 and labeled 20% of the verified subset as true or misclassified.

#### 2.2.2 Skewed‐Error Scenario

To evaluate the effect of skewed false‐positive rates on activity patterns and assess the efficacy of the proposed method at recovering correct patterns, we defined a case where a species follows a unimodal activity pattern while false positives clump in times of inactivity. For this case, we model the true activity pattern to adopt a single midday peak at π and false positives to cluster at night at 3π/2 rad. We kept all other settings identical to the uniform‐error scenario.

For each scenario, we discretized times into *J* = 24 bins, ran 4 Markov chains (1000 iterations, 500 warmup), and extracted posterior means and 95% credible intervals for the true activity histogram. Convergence was assessed when *R‐hat* ≤ 1.01 and effective sample size > 400. To quantify recovery of the true pattern, we computed the overlap coefficient Δ (Meredith and Ridout 2014) (Equation 3) across models and scenarios to evaluate the ability of each approach to correct for false positives in both uniform and skewed error situations.

(3) $$\Delta =\sum_{j=1}^{J}\min\left\{f_{1j},f_{2j}\right\}\mathrm{\Delta t}$$

where *f*_1*j*_ is the estimated activity in bin *j* derived from dataset 1. *f*_2*j*_ is the estimated activity in bin *j* derived from dataset 2.

#### 2.2.3 Correction Performance at Different False Positive Rates and Verification Effort

To quantify how much expert verification is needed for each correction model to recover the true activity pattern, we conducted a simulation analysis that calculates overlap values across corrected and true activity patterns for different combinations of activity pattern shapes, false positive rates and correction efforts. For every combination of (i) pattern shape namely, unimodal, bimodal or trimodal; (ii) false‐positive rate 0.05–0.30 (at 0.05, 0.10, 0.20, 0.30); (iii) verification effort 0.00–0.50 of records (at 0.05 intervals); and (iv) error model (uniform versus skewed), we simulated a dataset of *N* = 2000 detections. False positives were sampled either uniformly over the 24‐h cycle (for the uniform scenarios) or from a night‐centered distribution (3π/2 rad) to create structured error. Verified records were selected at random from the full dataset and their labels served as ground truth for model fitting. Each simulated dataset was analyzed with the matching model in RStan (1000 iterations, 500 warm‐up iterations over 4 chains). We extracted the posterior mean of the corrected 24‐bin histogram and compared it to the true histogram using the overlap coefficient.

To visualize the correction performance of our method, we drew the true, uncorrected, and corrected activity patterns and evaluated overlap (Δ) under each scenario. For the assessment, we consider a value of Δ = 1 as indicative of perfect recovery. For every scenario, we ran 1000 independent replicates, recorded the mean and standard deviation of Δ. We identified the minimum verification proportion required to achieve mean Δ ≥ 0.95. This design isolates how false positive rate, size of verified subset, and distribution of false positives across time of day jointly influence the fidelity of the corrected activity pattern curve.

### 2.3 Usage and Reproducibility

Decision by users on whether to use the uniform or skewed error distribution models can be made after examination of the temporal distribution of false positives in the expert‐verified subset of the data. Whilst the uniform‐error model is a subcase of the non‐uniform model, with the only difference being that errors in the former happen to be equal across time bins, the assumption of error distribution uniformity allows it to have fewer parameters, enhancing its performance when applied to smaller datasets.

### 2.4 Example Application

To illustrate practical utility of the method, we used data for three bird species: Cooper's hawk ( *Accipiter cooperii* ), the common nighthawk ( *Chordeiles minor* ) and the gray catbird ( *Dumetella carolinensis* ). Acoustic recordings were collected at the DeLuca Preserve, south‐central Florida (USA), using a grid of 48 SwiftOne autonomous recorders [Cornell Lab of Ornithology, Ithaca, New York, USA]. Audio was first processed with BirdNET (version 2.0.0, Kahl et al. 2021) using overlap = 0.5 and sensitivity = 1.4. Expert reviewers manually validated BirdNET‐labeled acoustic clips—Cooper's hawk (*n* = 395), common nighthawk (*n* = 807), and gray catbird (*n* = 899) classifying each detection as present (true positive) or absent (false positive). For each species, we plotted the uncorrected activity pattern using all BirdNET detections and the true pattern from all detections verified as present. To evaluate the correction model, we repeatedly (10 replicates) sampled a random 20% subset of detections to label as verified using human labels, fit the model using the verified and unverified detections, and compared the corrected and true activity patterns using temporal overlap. Consistent with common diel kernel density estimation workflows, we pooled detections across all 48 recorders to estimate a population‐averaged activity curve for each species. Under this pooled analysis, θ is interpreted as the dataset‐average true‐positive rate across the recorder grid, informed by the verified clips.

## 3 Results

### 3.1 Model Performance

The efficiency of the false positive activity model, in terms of the relative size of verified dataset required to achieve high accuracy, depended more on how false positives were distributed than on the shape of the underlying activity pattern (Table 1). When false positives were uniformly distributed across the day, the model was highly robust and needed minimal verification. For unimodal activity patterns no verification was needed at any of the tested false‐positive rates (0.05–0.30). Bimodal and trimodal patterns required a verified dataset that was at least 5% of the total dataset, irrespective of false positive rate, to meet the Δ ≥ 0.95 target.

**TABLE 1:** Minimum size of the human‐verified dataset (as % of the total dataset) required for reliable activity‐pattern correction (mean overlap, Δ ≥ 0.95).

False positive distribution	Activity pattern	False positive rate	Minimum size of verified dataset (% of total dataset)	Mean Δ	SD Δ
Uniform	Unimodal	0.05	0	0.988	0.002
		0.10	0	0.978	0.006
		0.20	0	0.962	0.009
		0.30	0	0.951	0.006
	Bimodal	0.05	5	0.989	0.002
		0.10	5	0.982	0.002
		0.20	5	0.973	0.006
		0.30	5	0.967	0.005
	Trimodal	0.05	5	0.988	0.002
		0.10	5	0.984	0.004
		0.20	5	0.973	0.005
		0.30	5	0.961	0.008
Skewed	Unimodal	0.05	0	0.972	0.002
		0.10	0	0.953	0.004
		0.20	15	0.959	0.013
		0.30	20	0.959	0.009
	Bimodal	0.05	0	0.967	0.003
		0.10	5	0.958	0.007
		0.20	15	0.965	0.009
		0.30	15	0.952	0.013
	Trimodal	0.05	5	0.975	0.004
		0.10	5	0.954	0.009
		0.20	10	0.954	0.014
		0.30	15	0.962	0.008

*Note:* We identified the minimum size of the human‐verified dataset required for different underlying activity pattern shapes (unimodal, bimodal, trimodal), simulated false positive rate, and distribution of false positives (uniform and skewed). We report the mean and SD overlap (Δ) achieved in each scenario across 1000 simulation replicates.

By contrast, skewed errors demanded larger verification datasets and the requirement scaled with error magnitude. When the false positive rate was < 0.10, a verified dataset that was at least 5% of the total dataset was sufficient for all three activity patterns. However, the minimum required size of the verification dataset increased to 10%–15% of the total dataset at a 0.20 false positive rate and to 15%–20% at a 0.30 false positive rate. Across all cases the overlap achieved at the minimum thresholds had a narrow range (mean Δ = 0.95–0.99; SD Δ ≤ 0.014), confirming that performance using the identified size of verified datasets was stable across replicates.

**FIGURE 1:**
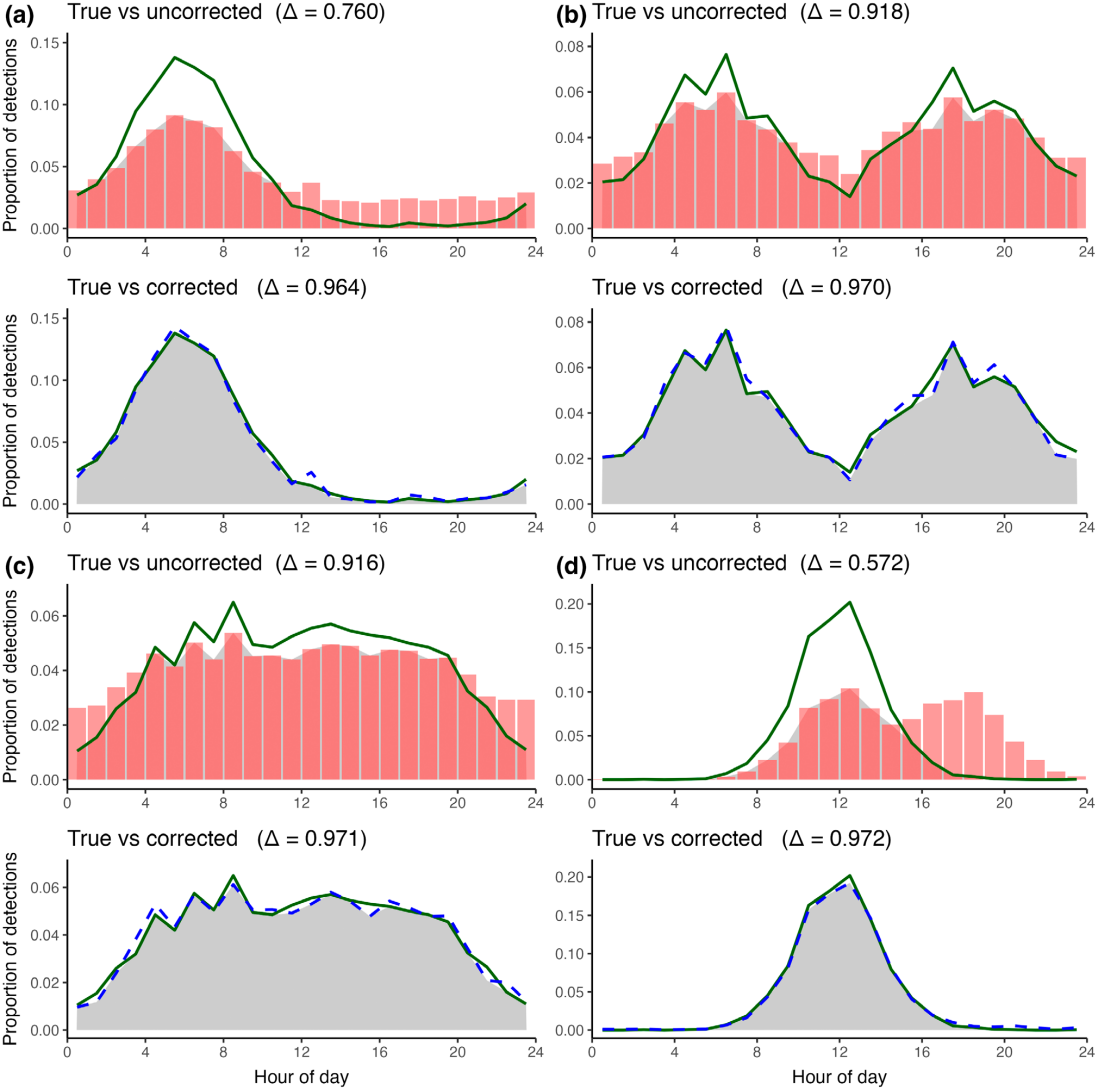
Performance of the Bayesian false‐positive‐correction models across four simulated scenarios. Panels show the true species activity pattern (green line), the uncorrected histogram (with false positives from misclassification; red bars), and the corrected curve for each scenario (blue dashed line); gray shading denotes the overlap between the two distributions being compared. In panels (a), (b), and (c) the false positives were uniformly distributed across time of day, and correction was performed using the uniform error model. Panel (a) has a true unimodal activity pattern, panel (b) has a true bimodal activity pattern, and panel (c) has a true trimodal activity pattern. Panel (d) has a true unimodal activity pattern with false positives skewed towards hours 16–24, fitted with the skewed error model. Hour of day is plotted on the *x* axis (0–24 h) and *y* axis shows the proportion of detections per 1‐h bin.

Across all simulated activity shapes, our Bayesian correction closely reproduced the underlying pattern, whereas the uncorrected activity pattern was visibly distorted by false positives (Figure 1). When misclassifications were uniformly distributed in time (unimodal, bimodal and trimodal scenarios; Figure 1A–C), the uncorrected activity patterns were relatively less biased (Δ = 0.76, 0.92, and 0.92, respectively) than when misclassifications had a skewed distribution (Figure 1D; Δ = 0.57). Nonetheless, our method further reduced the bias in activity patterns from misclassifications uniformly distributed in time and the corrected activity patterns overlapped the true curve almost perfectly irrespective of activity pattern shape (Δ = 0.96–0.97; Figure 1A–C).

The largest gain in overlap with the true activity patterns between uncorrected and corrected activity patterns occurred when false positives were temporally clustered (night‐skewed scenario; Figure 1D). Here, the uncorrected distribution showed an artificial nocturnal peak that overlapped little with the true activity pattern (Δ = 0.57). After correction, the curve realigned with the midday peak of the true distribution obtaining high overlap (Δ = 0.97, an increase in Δ of 0.40; Figure 1D).

### 3.2 Example Application

Human experts identified varying levels of false positives across the target species. These amounted to 34.7% of Cooper's hawk BirdNET detections, 19.5% for the gray catbird and 17% for the common nighthawk. Accordingly, overlap between the true and uncorrected activity patterns was lowest for Cooper's hawk (79.7%), high for common nighthawk (93.2%), and highest for gray catbird (97.4%). After applying the correction, overlap with the true pattern increased to 90.0% (SD = 2.4%) for Cooper's hawk and to 95.7% (SD = 1.1%) for common nighthawk, while gray catbird showed a slight mean decrease to 96.8% (SD = 0.6%).

## 4 Discussion

The Bayesian hierarchical modeling approach we proposed addressed false positive induced bias in diel activity pattern analyses. It complements the growing body of false‐positive‐informed methods developed for other ecological inference targets. In particular, false positives have been explicitly modeled in occupancy analyses (e.g., Miller et al. 2011; Clement et al. 2022) and in abundance estimation using N‐mixture and related frameworks, including recent approaches that integrate automated acoustic detections with auxiliary data to account for misclassification and detection heterogeneity (e.g., Chambert et al. 2016; Doser et al. 2021; Udell et al. 2024). Together, these approaches contribute to an expanding toolkit for correcting false positives across ecological workflows.

Our results demonstrate that accounting for false positives improves the accuracy of diel activity inference and can substantially reduce the amount of manual verification needed relative to fully expert‐labeled datasets. Most notably, when misclassifications are concentrated at particular times of day, they can introduce pronounced false peaks and apparent changes in activity that are artifacts of the classification process rather than genuine behavioral patterns.

In the empirical data example, species‐specific false‐positive rates from BirdNET produced measurable differences in diel activity curves. Cooper's hawk, the species with the highest misclassification, showed the lowest overlap between the human‐verified and uncorrected patterns, whereas the species with relatively few false positives (gray catbird) was less affected. Partial verification (20% of the dataset) substantially increased overlap for Cooper's hawk and modestly improved those of the common nighthawk, indicating that gains scale with the magnitude of misclassification. The slight decrease in overlap for the gray catbird is also expected as random human auditing when false positives are rare can artificially inflate the model's estimate of the misclassification rate and lead to overcorrection. As the volume of automatically collected and labeled ecological data quickly surpasses the capacity for full human verification, these results support a hybrid workflow for wildlife monitoring combining machine learning and human auditing. Researchers can allocate more human verification effort towards taxa with higher error rates for which our method has demonstrated benefits. For species with limited misclassifications, direct plotting of uncorrected data will likely produce reliable activity patterns.

As our method is meant to correct population‐level diel activity patterns through similar workflows to the currently widely adopted kernel density estimators, the main described use‐case focuses on diel heterogeneity in false‐positive distribution and does not explicitly model site level heterogeneity. In this context, Iannarilli et al. (2025) recently called for hierarchical diel activity estimators that can accommodate spatial structure and repeated measures. Consistent with common kernel workflows, our method pooled detections for each species to estimate a single, population‐averaged activity curve. Therefore, θ is interpreted as the average true‐positive rate for the analyzed dataset learned from the full verified subset.

Misclassification prevalence may vary across sites or other covariates. Crucially, the direct mechanism by which false positives distort diel activity curves is typically temporal structure in misclassifications (i.e., errors concentrated at times of day). Our model addresses this by learning the false‐positive time‐of‐day distribution directly from the verified subset, yielding an estimated diel false‐positive distribution *f*_false_. A hierarchical θ formulation is most beneficial when a subset of sites contributes disproportionately to false positives and that contribution is not well represented in the verified subset and/or is associated with a different time‐of‐day error profile than the remainder of the dataset. More broadly, a hierarchical θ formulation should be used when it is suspected that there could be meaningful heterogeneity in θ across space or time. In such cases, a single pooled θ may not represent all sampling units equally and could bias inference if the verified subset is not representative of that heterogeneity. When users suspect such variation, we recommend stratified verification across relevant covariates and a hierarchical extension in which θ is modeled with a logit link and covariates and/or random effects.

Our framework is complementary to modern false‐positive occupancy and N‐mixture approaches in that both rely on a mixture formulation to estimate the probability that an apparent detection is truly the focal species (i.e., a true‐positive rate). In occupancy/abundance settings, heterogeneity in this probability is often driven by spatial and temporal variation in underlying abundance and detection conditions, and these models leverage replicated sampling structures to estimate such heterogeneity and propagate it into inference on species distributions and estimates. Future work could formally integrate our diel correction approach into false‐positive occupancy/abundance models by adding an explicit time‐of‐day component to the observation/misclassification process. In such joint model, replicated site/night data could provide stronger information to estimate θ (and its covariate‐driven variation), while the diel mixture components (*f*_true_ and *f*_false_) could use time‐of‐day information to better separate true detections from temporally structured false positives. This integration could improve ecological inference in monitoring programs where automated classification error and activity shifts co‐vary over space, season, or time.

## Author Contributions

**Mohamed Khalil Meliane:** conceptualization (lead), data curation (equal), formal analysis (lead), writing – original draft (lead), writing – review and editing (equal). **Nicole Rita:** data curation (equal), investigation (equal), writing – review and editing (equal). **Zachery B. Holmes:** data curation (equal), investigation (equal), writing – review and editing (equal). **Joseph M. Guthrie:** funding acquisition (equal), investigation (equal), project administration (equal), resources (equal), validation (equal), writing – review and editing (equal). **E. Hance Ellington:** data curation (equal), funding acquisition (equal), investigation (equal), methodology (equal), project administration (equal), resources (equal), supervision (lead), validation (lead), writing – review and editing (equal).

## Funding

This work was supported by Division of Biological Infrastructure (Grants 0735191, 1265383, 1743442, and 2153040); Live Wildly Foundation; National Fish and Wildlife Foundation (grant 80416), National Institute of Food and Agriculture (Grant 1026189) and Bellini Better World.

## Conflicts of Interest

The authors declare no conflicts of interest.

## Data Availability

The code and data used in this manuscript are publicly available on https://doi.org/10.5281/zenodo.19052420.
